# Large area single crystal gold of single nanometer thickness for nanophotonics

**DOI:** 10.1038/s41467-024-47133-7

**Published:** 2024-04-02

**Authors:** Chenxinyu Pan, Yuanbiao Tong, Haoliang Qian, Alexey V. Krasavin, Jialin Li, Jiajie Zhu, Yiyun Zhang, Bowen Cui, Zhiyong Li, Chenming Wu, Lufang Liu, Linjun Li, Xin Guo, Anatoly V. Zayats, Limin Tong, Pan Wang

**Affiliations:** 1 Interdisciplinary Center for Quantum Information, New Cornerstone Science Laboratory, State Key Laboratory of Extreme Photonics and Instrumentation, College of Optical Science and Engineering, Zhejiang University, Hangzhou, 310027 China; 2 Interdisciplinary Center for Quantum Information, State Key Laboratory of Extreme Photonics and Instrumentation, ZJU-Hangzhou Global Scientific and Technological Innovation Center, Zhejiang University, Hangzhou, 310027 China; 3 Department of Physics and London Centre for Nanotechnology, King’s College London, Strand, London WC2R 2LS UK; 4 Jiaxing Key Laboratory of Photonic Sensing & Intelligent Imaging, Jiaxing, 314000 China; 5 Intelligent Optics & Photonics Research Center, Jiaxing Research Institute Zhejiang University, Jiaxing, 314000 China; 6 Collaborative Innovation Center of Extreme Optics, Shanxi University, Taiyuan, 030006 China

**Keywords:** Optical materials and structures, Nanophotonics and plasmonics, Nanoscale materials

## Abstract

Two-dimensional single crystal metals, in which the behavior of highly confined optical modes is intertwined with quantum phenomena, are highly sought after for next-generation technologies. Here, we report large area (>10^4^ μm^2^), single crystal two-dimensional gold flakes (2DGFs) with thicknesses down to a single nanometer level, employing an atomic-level precision chemical etching approach. The decrease of the thickness down to such scales leads to the quantization of the electronic states, endowing 2DGFs with quantum-confinement-augmented optical nonlinearity, particularly leading to more than two orders of magnitude enhancement in harmonic generation compared with their thick polycrystalline counterparts. The nanometer-scale thickness and single crystal quality makes 2DGFs a promising platform for realizing plasmonic nanostructures with nanoscale optical confinement. This is demonstrated by patterning 2DGFs into nanoribbon arrays, exhibiting strongly confined near infrared plasmonic resonances with high quality factors. The developed 2DGFs provide an emerging platform for nanophotonic research and open up opportunities for applications in ultrathin plasmonic, optoelectronic and quantum devices.

## Abstract (web summary)

2D metallic single crystals are sought after for nanophotonic applications, but their synthesis remains challenging. Here, the authors report an atomic level precision etching method to fabricate large-area crystalline gold flakes with nanometre thickness, showing enhanced plasmonic and nonlinear optical properties.

## Introduction

Low-dimensional metals have attracted extraordinary research interest because of their unconventional physical, chemical, and mechanical properties arising from the reduced dimensionality^1–4^. Among them, ultrathin (few-nanometer thick) two-dimensional (2D) gold, which combines unique advantages of quantum effects in its electric and optical properties^5–8^, plasmon-enabled extreme (nanometer scale) light confinement^9–12^, high optical transparency and excellent chemical stability, is highly desired for the enhancement of light–matter interaction at the nanoscale for the state-of-the-art nanophotonic research and the realization of next-generation ultrathin plasmonic, optoelectronic, photonic and quantum devices^6,9–11,13–15^. To date, various wet-chemical approaches, such as seed-mediated synthesis^16–20^, polyol reduction method^21–24^, 2D template-directed synthesis^25–32^, methyl orange-assisted synthesis^33,34^ and others^35–37^, have been developed for the fabrication of 2D gold with high crystalline quality and thicknesses down to a sub-nanometer scale^29,33^. However, due to the proportional increase in the thickness and lateral size with the growth time, it is very challenging to fabricate ultrathin 2D gold with a large area. For example, for 2D gold with a sub-5-nm thickness, the lateral size is usually at a sub-micrometer scale^20,25,29,30,32,33,36^, which imposes restrictions for most applications. Moreover, it is difficult to precisely control the thickness of 2D gold with wet-chemical approaches, while it is highly desired due to the high sensitivity of its electric and optical properties to the thickness because of the quantum-confinement or electron surface scattering effects^5,6,11,14^. Ultrathin gold films on dielectric substrates have recently been obtained with deposition approaches using adhesive/seeding layers, such as metals (e.g., copper)^11,14,38^ and organosilane monolayers^39^, to reduce the percolation threshold of gold. However, as-fabricated ultrathin gold films have a granular polycrystalline structure that can affect their performance in many applications (e.g., due to the electron scattering losses introduced by surface roughness and grain boundaries^40–43^). In addition, it is difficult to detach them from the substrates due to the existence of adhesion/seeding layers, which greatly limits their flexibility for fundamental studies and applications (e.g., integration with other structures and devices).

Here, we introduce an atomic-level precision etching (ALPE) approach to circumvent the lateral size-thickness relation in wet-chemical approaches, enabling the fabrication of large area (>10^4^ μm^2^) single crystal 2D gold flakes (2DGFs) with thicknesses down to a single nanometer level. The dramatic decrease of the gold thickness down to such scales while keeping a single crystal structure and a smooth surface not only endows 2DGFs with quantum-confinement-augmented optical nonlinearity (e.g., more than two orders of magnitude enhancement in harmonic generation), but also provides a promising platform for the realization of low-loss plasmonic nanostructures with extreme optical confinement, as demonstrated by patterning 2DGFs into nanoribbon arrays exhibiting near infrared plasmonic resonances with high quality factors.

## Results

### Fabrication and characterization of 2DGFs

**Fig. 1:**
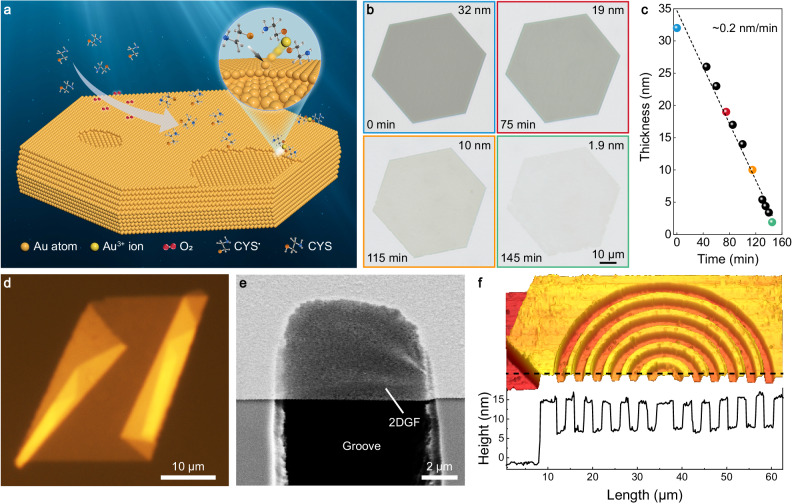
Fabrication of two-dimensional gold flakes (2DGFs). **a** Schematic illustration of the atomic-level precision etching approach for fabricating 2DGFs. The light blue arrow shows the reaction of cysteamines with oxygen molecules to form cysteamine radicals (CYS cysteamine, CYS^•^ cysteamine radical). Inset, enlarged view of the etching of gold atoms with cysteamine radicals to form a soluble gold-thiolate complex. **b** Optical transmission micrographs of a gold flake taken at various etching times. **c** Measured thickness of the gold flake as a function of the etching time. The dashed line is a linear fit to the measured data points. **d** Optical reflection micrographs of a 2DGF folded on itself. **e** Scanning electron microscopy image of a 3.8-nm-thick 2DGF suspended across a groove. **f** Atomic force microscopy (AFM) image of a gold flake locally etched to implement a concentric ring pattern. A line scan along the indicated dashed line is also shown.

Large-area single crystal gold flakes (tens of nanometers in thickness, Supplementary Fig. 1) were firstly synthesized on a substrate (e.g., mica with an atomically smooth surface)^23,43^ as the starting structures. Then, they were immersed into a cysteamine solution (200 mM in chloroform, the replacement of commonly used water^44^ with chloroform as the solvent is beneficial for the uniform and controllable etching of gold over macroscopically large areas) to initiate chemical etching. During this process, gold atoms on the surface of the gold flakes are etched by cysteamine radicals (produced via the reaction of cysteamines with dissolved oxygen molecules in the solution) via an oxidation-reduction process to form soluble gold-thiolate complexes^44,45^. Benefitting from their single crystal structure, the gold flakes can be gradually etched one atomic layer after another to form substrate-supported 2DGFs, as schematically shown in Fig. 1a. In contrast, it is difficult to obtain ultrathin gold films by chemical etching of polycrystalline gold films (Supplementary Fig. 2) due to the difference in the etching rate for gold with different crystalline facets (there exist small differences in their surface energies, leading to the difference in their stability and reactivity)^44^. Figure 1b presents optical micrographs of the etching of a gold flake with its thickness decreasing from 32 to 1.9 nm (accompanied by a visible increase in transmission), while its lateral size remains almost unchanged. It is worth noting that the chemical etching takes place also at the edge of the gold flakes (which has a different crystalline facet from that of the top and bottom surfaces^23^ and, therefore, different etching rate), but it has no observable effects on the shape and lateral size of the etched gold flakes (Supplementary Fig. 3), which is critical for the fabrication of 2D gold with a large area. The etching rate was estimated to be ~0.2 nm/min (Fig. 1c) and is dependent on the concentration of the cysteamine. This indicates the crucial ability to control the thickness of the 2DGF at an atomic-level precision, which is difficult for conventional wet-chemical approaches and is of great importance for the precise engineering of properties of the 2DGF^5,6,11,14^.

The lateral size of the 2DGF is determined by the size of the initial gold flakes, which can be at a 100s-μm scale (Supplementary Fig. 4). Its area is at least five orders of magnitude larger than that of 2D gold of similar thicknesses obtained with other methods^25,30^. Such a large area makes it possible to exploit 2DGFs for applications in fundamental studies and the construction of ultrathin metal-based structures and devices. Because of the absence of adhesive layers, as-fabricated 2DGFs can be easily detached from the substrate, as evidenced by the optical micrograph of a 2.8-nm-thick 2DGF folded on itself (Fig. 1d). Therefore, similar to other 2D materials, 2DGFs with such a large area can be readily manipulated and transfer-printed for integration with other materials and structures (see Methods and Supplementary Fig. 5), greatly expanding their flexibility for fundamental studies and applications. For example, a 2DGF can be transfer-printed to be suspended over a groove (Fig. 1e) or onto a curved sidewall of an optical microfiber (Supplementary Fig. 6a), either of which is difficult to realize with conventional deposition approaches. It can also be transfer-printed sequentially to form a stacked multilayer structure (Supplementary Fig. 6b) or onto a WS_2_ monolayer to form an ultrathin metal-semiconductor heterostructure (Supplementary Fig. 6c). Compared to conventional deposition approaches, the transfer printing of 2DGFs provides a gentle and damage-free approach for metal integration, which is especially attractive for delicate materials such as 2D semiconductors and organic molecules^46^. Furthermore, a 2DGF can be rolled up into a microtube structure with a diameter of ~2 μm (Supplementary Fig. 6d)^47^, showing its excellent mechanical flexibility and the potential for introducing a strain to further engineer its electric and optical properties^48^.

The ALPE approach can be applied to spatially localized areas to create micro/nanostructures (see Methods and Supplementary Fig. 7). As an example, Fig. 1f shows an atomic force microscopy (AFM) image of a gold flake locally etched to implement a concentric ring pattern (see Supplementary Fig. 8a for its optical micrograph), whose thickness is about 10 nm less than that of the surrounding area (see Supplementary Fig. 8b, c for the cross-sectional TEM image of the etched edge). The ALPE approach can also be applied to the fabrication of 2D silver and copper flakes (Supplementary Fig. 9), showing the applicability of this approach to other metals.

**Fig. 2:**
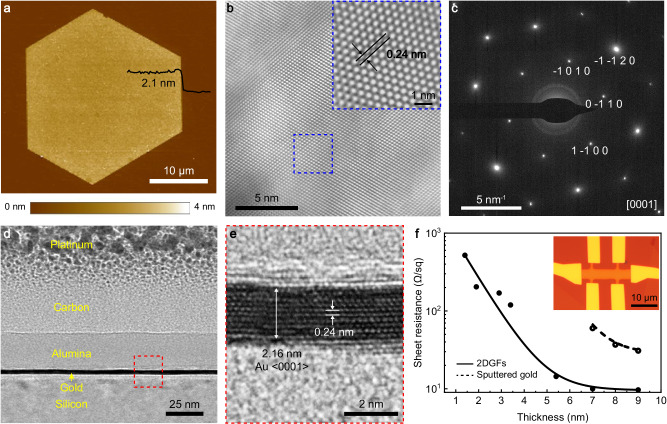
Structural and electric properties. **a** AFM image of a 2.1-nm-thick 2DGF. The black line shows the height profile of the edge of the 2DGF in the specified area. **b** Planar transmission electron microscopy (TEM) image of a 3.7-nm-thick 2DGF. Inset, enlarged view of the marked region. **c** Electron diffraction pattern of the region marked in (**b)**. **d** Cross-sectional TEM image of a 2DGF. **e** Atomic-resolution TEM image of the region marked in (**d**). **f** Thickness-dependent sheet resistance of 2DGFs and sputtered gold films (the latter is conductive only for thicknesses larger than the percolation thickness of ~7 nm). The solid and dashed lines are exponential fits to the measured data points. Inset, optical micrograph of a Hall-bar structure fabricated with a 5.4-nm-thick 2DGF.

AFM and transmission electron microscopy (TEM) were used to characterize the surface morphology and crystalline structure of as-fabricated 2DGFs. The AFM image of a typical 2DGF (Fig. 2a) shows that it has a thickness of 2.1 nm and a smooth surface with a root-mean-square roughness (RMS) of ~0.25 nm (measured over an area of ~300 μm^2^, see also Supplementary Fig. 10 for AFM images taken from three different locations on the 2DGF with an area of ~0.01 μm^2^, which give a better RMS roughness of ~0.15 nm). This reveals the uniform atomic monolayer-by-monolayer etching characteristic of the approach that can retain the excellent surface quality and thickness uniformity of the initial flake, which is important for obtaining large-area 2DGFs. The direct etching of gold flakes on a substrate is also critical for obtaining 2DGFs with an excellent surface smoothness across the whole area, which provides a method to avoid wrinkles encountered by 2D metals prepared with wet-chemical approaches during their deposition onto a substrate^19,28,31,35,37^. The high-resolution TEM images of the surface of a 3.7-nm-thick 2DGF (Fig. 2b) show highly periodic lattice fringes with an interplanar spacing of ~0.24 nm (inset). The single crystal property of the 2DGF was confirmed by the electron diffraction pattern (Fig. 2c), showing a hexagonal close-packed structure with a <0001> crystal orientation. Figure 2d (and Supplementary Fig. 11) further presents a cross-sectional TEM image of a 2DGF (see Methods for details), in which 10 atomic planes of gold can be clearly observed (Fig. 2e). 2DGFs with such a large area is stable under ambient conditions for at least 6 months (Supplementary Fig. 12).

The excellent structural quality of 2DGFs is further confirmed by the investigation of their electric properties using a four-probe approach based on a Hall-bar structure (inset of Fig. 2f, see Methods and Supplementary Fig. 13 for details). With the decrease of the thickness from 9 to 1.4 nm, the sheet resistance of 2DGFs increases from ~9 to 530 Ω per square (Ω/sq) (solid dots, Fig. 2f). By contrast, sputtered gold films are only conductive for thicknesses larger than the percolation threshold of ~7 nm, their sheet resistance increases quickly from ~31 to 61 Ω/sq when the film thickness decreases from 9 to 7 nm (hollow dots, Fig. 2f). The pronounced decrease in the sheet resistance of 2DGFs is due to the greatly reduced electron scattering losses in 2DGFs with high crystal quality and excellent surface smoothness. Therefore, 2DGFs provide a promising platform for nanophotonic applications with minimized intrinsic loss.

### Linear optical properties of 2DGFs

**Fig. 3:**
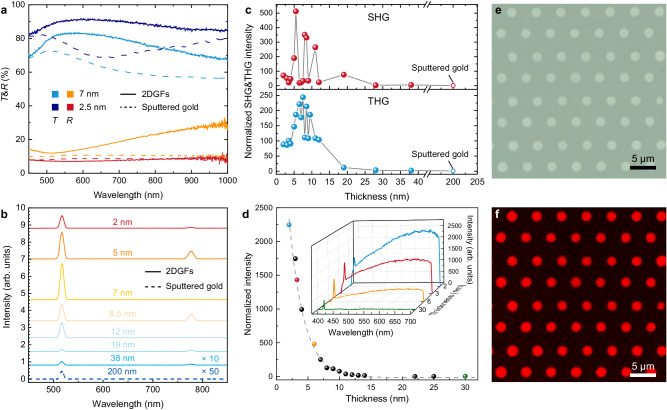
Thickness-dependent optical properties. **a** Measured transmittance (*T*) and reflectance (*R*) of 2DGFs (solid lines) and sputtered gold films (dashed lines) with different thicknesses on a mica substrate. **b**, **c** Nonlinear emission spectra measured from 2DGFs with various thicknesses and a 200-nm-thick sputtered gold film (**b**) and the thickness dependence of second-harmonic generation (SHG) and third-harmonic generation (THG) intensities from the 2DGFs normalized with those from the 200-nm-thick sputtered gold film (**c**) under *p*-polarized pulsed laser excitation (1550-nm wavelength, ~140-fs pulse width) at an incident angle of 30°. The spectra in (**b**) are shifted along the vertical axis for visibility. **d** Thickness-dependent multiphoton photoluminescence (MPPL) intensity from 2DGFs obtained under *p*-polarized pulsed laser excitation (800-nm wavelength, ~100-fs pulse width) at an incident angle of 30°. The inset shows the measured emission spectra from 2DGFs with thicknesses of 2, 3, 6, and 30 nm. The dashed line is an exponential fit to the measured data points. **e**, **f** Optical transmission micrograph (**e**) of a microhole array locally etched in a 24-nm-thick gold flake (the height contrast is 15 nm) and its MPPL image (**f**) with the signal integrated over a 575–630 nm spectral range.

The decrease in the thickness endows 2DGFs with intriguing optical properties. Transmission and reflection spectra of 2DGFs with different thicknesses were compared to sputtered gold film counterparts (Fig. 3a). Due to the presence of electrically unconnected gold islands in the investigated sputtered gold films, a dip (peak) in transmittance (reflectance) around 650 nm, that red-shifts and widens with increasing thickness, can be observed (dashed lines) as a signature of the excitation of localized surface plasmon (LSP) modes of the gold islands^11^. By contrast, these dips/peaks are absent in the transmission and reflection spectra for 2DGFs with thickness down to 2.5 nm (solid lines), further indicating the excellent continuity of 2DGFs. Benefitting from the crystalline quality and surface smoothness, 2DGFs have a much higher transmittance compared with that of sputtered gold films with the same thickness. Particularly, for the 2.5-nm-thick 2DGF, the transmittance around 600 nm reaches a value of ~91%, making it an attractive material for transparent and flexible electrodes in optoelectronic devices.

### Nonlinear optical properties and quantum effects

As the thickness of 2DGFs approaches a few nanometers, quantization of the electronic energy in the out-of-plane direction becomes important^5^ (Supplementary Fig. 14), which makes a crucial impact on the nonlinear optical response of 2DGFs^6,15,49,50^. Under pulsed laser excitation at 1550-nm wavelength (see Methods and Supplementary Fig. 15), the nonlinear emission spectra from both 2DGFs with various thicknesses and a 200-nm-thick sputtered gold film feature narrow peaks at 775 and 516.7 nm (Fig. 3b), which corresponds to the second-harmonic generation (SHG) and third-harmonic generation (THG) signals, respectively. The SHG and THG intensities from the 2DGFs are much higher than those from the sputtered gold film, and are strongly dependent on the thickness. With the decrease of the thickness, they increase nonmonotonously, exhibiting very sharp oscillations with thickness (Fig. 3c). The SHG and THG intensities are enhanced by ~500 and 250 times for the 2DGFs with thicknesses of 5 and 7 nm, respectively. This can be explained by quantization of the electronic energy levels of the 2DGFs so that the optical transitions excited by photons with fixed energy between the intersubband levels are in and out of resonance as the thickness changes, resulting in the resonant enhancement^6,49–51^. The resonances occur at different thicknesses of gold for SHG and THG processes and agree well with the simulation results (Supplementary Fig. 16a, b) based on the quantum electrostatic model^6^ (see Methods). It is worth noting that the measured thickness-dependent THG is observed on a broad underlying background, which can be attributed to the contribution from interband transitions in the 2DGFs with quantized electronic states^49^ (see Methods and Supplementary Fig. 16c). The SHG intensity from 2DGFs under the excitation with 800-nm laser pulses (Fig. 3d, inset) also exhibits the thickness-dependent oscillatory behavior (Supplementary Fig. 17). In addition to the SHG signal, broad multiphoton photoluminescence (MPPL) background is also observed, identified by typical excitation power dependences (Supplementary Fig. 18). With the decrease of the gold thickness from 30 to 2 nm, the spectrally integrated MPPL intensity increases by ~2200 times (Fig. 3d). The enhancement is so high because in bulk gold MPPL is a very inefficient process, as the momentum of the photon is too small to satisfy momentum conservation of the involved intraband transition in the *sp* conduction band^52^. Benefiting from the thickness-dependent quantization of the energy levels, intersubband transitions in 2DGFs do not require an additional momentum, which greatly boosts the MPPL efficiency^52^. Therefore, single crystal 2DGFs with significant quantum-confinement-augmented optical nonlinearity provide an emerging material for nonlinear optical research and applications.

Due to the strong thickness dependence of the nonlinear optical properties, the ALPE approach can be used to locally engineer the optical nonlinearity of gold flakes. As an example, a microhole array was etched into a 24-nm-thick gold flake (Fig. 3e), with its thickness locally modified with a 15-nm step. Under the excitation with 800-nm laser pulses, the microhole array was observed in the MPPL image taken with a 575–630 nm bandpass filter as a red emission pattern on a completely dark background (Fig. 3f), revealing significantly enhanced MPPL in the thinner microhole array region compared to a thicker surrounding. This is particularly important for applications in which high local optical nonlinearity is required without the integration of other, e.g. technologically incompatible, materials.

### Low-loss nanoplasmonics

**Fig. 4:**
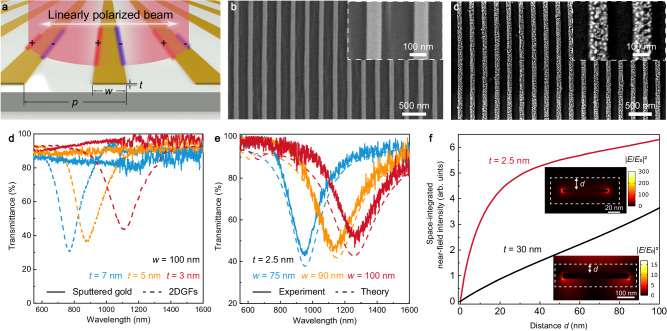
2DGFs for low-loss nanoplasmonics. **a** Schematic illustration of patterning of a 2DGF into a nanoribbon array supporting dipolar localized surface plasmon (LSP) modes. Parameters *w*, *p*, and *t* represent the width, period, and thickness of a nanoribbon, respectively. **b**, **c** Scanning electron microscopy (SEM) images of nanoribbon arrays fabricated from a 2DGF (**b**) and a sputtered gold film (**c**) with the same thickness of 3 nm. Insets, enlarged SEM images of the nanoribbons. **d** Transmittance of nanoribbon arrays fabricated with 2DGFs (dashed lines) and sputtered gold films (solid lines) for various nanoribbon thicknesses (*w* = 100 nm). **e** Measured (solid lines) and calculated (dashed lines) transmittance of nanoribbon arrays fabricated with 2DGFs for various nanoribbon widths (*t* = 2.5 nm). **f** Numerically calculated space-integrated near-field intensities confined within an area extending by a distance *d* outside the nanoribbons made from a 2.5-nm-thick 2DGF and a 30-nm-thick sputtered gold film. The insets show the corresponding near-field intensity distributions of the nanoribbons having the same resonance wavelength of 970 nm.

The nanometer-scale thickness of 2DGFs, together with their 100s-μm lateral size, excellent crystal quality, and smooth surface, also enables the realization of low-loss plasmonic nanostructures with extreme optical confinement^9,11,12,53,54^. To implement a typical plasmonic nanostructure supporting LSP resonances, 2DGFs were patterned into nanoribbon arrays using the local etching approach (Fig. 4a). First, nanoribbon arrays with a fixed width (*w* = 100 nm) and period (*p* = 3*w*) were fabricated using 2DGFs with thicknesses of 7, 5, and 3 nm. Figure 4b shows an example of an as-fabricated nanoribbon array with a thickness of 3 nm, featuring a smooth surface and having no visible defects. In marked contrast, for nanoribbon arrays fabricated using sputtered gold films (see Methods), the nanoribbons are quite rough and become discontinuous when the thickness is less than ~7 nm (Fig. 4c). As a result, there is no observable LSP mode in their transmission spectra (Fig. 4d, solid lines); however, strong resonance dips due to the excitation of a dipolar LSP mode in each of the nanoribbons can be observed for the nanoribbon arrays fabricated using 2DGFs (Fig. 4d, dashed lines). The resonance dip is red-shifted dramatically from 767 to 1115 nm with the decrease of the nanoribbon thickness from 7 to 3 nm by only 4 nm, showing a large tunability of the plasmonic response as a consequence of the ultrathin thickness. This also indicates the importance of the sub-nanometer-level thickness control for ultrathin plasmonic structures, which can be easily realized with the simple ALPE approach (Fig. 1c). The quality factor for the plasmonic mode of the nanoribbon array with 3-nm thickness is up to ~5, which can be attributed to the greatly reduced electron scattering losses in the smooth single crystal 2DGFs (as evidenced by the excellent structural and electric properties presented in Fig. 2)^11,53^. The resonance wavelength of the plasmonic modes in the ultrathin nanoribbons can also be tuned by changing their width (Fig. 4e, solid lines). With the decrease of the nanoribbon width from 100 to 75 nm (*t* = 2.5 nm, *p* = 3*w*), the resonance dip is blue-shifted from 1264 to 951 nm. Numerically simulated transmission through the gold nanoribbon arrays (see Methods) confirms the experimental results (Fig. 4e, dashed lines).

Despite similar plasmonic resonances can be obtained in nanoribbon arrays fabricated using sputtered gold films with larger thicknesses (e.g., 30 nm, see Supplementary Fig. 19), nanoribbon arrays with an ultrathin thickness enabled by the 2DGFs, as shown in Fig. 4f, provide attractive advantages including tighter optical field confinement around the nanoribbon (cf., red and black lines), larger local-field enhancement (~20 times higher) and smaller footprint (~5 times reduction in width). Compared with plasmons in graphene and other 2D semiconductors, which usually lie at mid-infrared frequencies^55^, 2DGFs with a much higher free electron density allow the realization of strongly confined plasmons operating in the technologically appealing near infrared spectral range^11,53^. Large-area single crystal 2DGFs, merging low-loss strongly confined plasmons with the quantum-confinement-augmented optical nonlinearity, allow access to the regime of nanoscale light–matter interactions for fundamental studies^9,12,15,54^ as well as the realization of ultrathin nanophotonic devices including ultrafast optical modulators^10,11^ and high-sensitivity optical sensors^55^.

## Discussion

Using an ALPE approach, we have successfully fabricated 2D single crystal gold with lateral sizes extending over a 100s-μm scale and thicknesses down to a single nanometer level. The thickness of 2DGFs can be further pushed down to a sub-nanometer level by optimizing the etching conditions (Supplementary Fig. 20). Excellent single crystal quality of the ultrathin 2DGFs, together with the possibility of nanopatterning and ease of transfer printing, enables the realization of truly nanoscale photonic structures and devices with substantially decreased loss. Owing to their excellent plasmonic response, quantum-confinement-augmented optical nonlinearity, high transparency, and transferability, 2DGFs provide an emerging platform for fundamental research in various disciplines, such as physics, electronics, chemistry, and mechanics, and promise many unique opportunities in the development of ultrathin plasmonic, optoelectronic, photonic and quantum devices.

## Methods

### Transfer printing of 2DGFs

A polydimethylsiloxane (PDMS)-assisted transfer printing approach was used to pick up a 2DGF from a substrate and transfer print it onto targeted substrates or structures. As schematically shown in Supplementary Fig. 5, to facilitate the pick up of the 2DGF from the substrate, water vapor from water heated in a vial was first evaporated onto a small patch of PDMS to form numerous micrometer scale water droplets. Secondly, under an optical microscope, the PDMS film was quickly aligned (done within a minute to avoid the evaporation of the water droplets) and pressed onto the targeted 2DGF, and then it was slowly withdrawn backward to pick the 2DGF up. Finally, the 2DGF attached to the PDMS film was aligned and pressed into full contact precisely with the targeted substrate or structure with a three-dimensional positioning stage. The 2DGF was left on the substrate or the structure after a careful withdrawal of the PDMS film. The picking-up and depositing procedures were all conducted at room temperature.

### Local etching of gold flakes

To locally etch a gold flake, electron beam lithography (EBL) was used to precisely define the pattern with a resist mask and then the ALPE approach was applied to etch the exposed region of the gold flake. The process for the local etching is schematically shown in Supplementary Fig. 7. Firstly, a 200-nm-thick resist (positive resist PMMA 950 K or negative resist ma-N 2401) was spin-coated onto the substrate with a targeted gold flake present on it. Secondly, to ensure a good conductivity of the sample for the EBL, a conductive layer (AR-PC 5090) with a thickness of 90 nm was additionally spin-coated on the resist. Thirdly, the resist on the targeted gold flake was exposed with an electron beam under an acceleration voltage of 30 kV (Raith 150 Two, Raith) to write a designed pattern, which was followed by a developing process after removing the conductive layer with water. After that, the sample was immersed into an aqueous solution of cysteamine (25 mM in concentration), in which the exposed region of the gold flake can be precisely etched by controlling the etching time. It is worth noting that, for the local etching, water was used as the solvent to avoid the dissolution of the resist mask by chloroform. Finally, the sample was immersed into acetone to remove the resist and dried with nitrogen.

### TEM characterization

Planar TEM images were acquired with a Talos F200X G2 high-resolution transmission electron microscope operated at 200 kV. To prepare TEM grids with the samples, a 2DGF was first fabricated on a substrate using the ALPE approach, and then a fraction of the 2DGF was picked up from the substrate and transferred onto a copper grid by a fiber taper^56^. For the cross-sectional TEM characterization of 2DGFs, an electron-transparent cross-sectional lamella of the sample was prepared as follows. Firstly, a 2DGF was fabricated on a silicon substrate using the ALPE approach, followed by the deposition of a layer of Al_2_O_3_ (~30 nm in thickness) onto it using atomic layer deposition (SENTECH SI ALD) at 100 °C^43^. Secondly, layers of carbon (~50 nm in thickness) and platinum (~70 nm in thickness) were further sputtered on the sample to protect it. Then, a cross-sectional lamella of the sample with a thickness of less than 50 nm was obtained using a focused-ion-beam system (Helios G4, Thermo Scientific). Finally, the lamella was transferred onto a copper grid, and imaged using a Talos F200X G2 high-resolution transmission electron microscope operated at 200 kV.

### Measurement of sheet resistance

A four-probe approach based on a Hall-bar structure was used to investigate the electrical properties of 2DGFs (as schematically shown in Supplementary Fig. 13). After the fabrication of a 2DGF on a 300-nm-thick silica-coated silicon substrate using the ALPE approach, the local etching approach was used to pattern the 2DGF into the Hall-bar structure. Then, EBL was used to fabricate a mask for the deposition of metal contact electrodes (Cr/Au: 5/80 nm), followed by a lift-off process. An optical microscopy image of an as-fabricated Hall-bar structure is shown in the inset of Fig. 2f. The samples for sputtered gold films were fabricated as follows. EBL was first used to define masks of the Hall-bar structures on a 300-nm-thick silica-coated silicon substrate. Secondly, to avoid the use of metallic adhesion layers (such as Cr, Ti) that can affect the electrical property of the gold films, the exposed region of the masks was functionalized with a monolayer of (3-aminopropyl)trimethoxysilane instead before the deposition of gold^43^. Then, gold films with various thicknesses were deposited on the substrates under a base pressure of ~5 × 10^−6^ Torr at a rate of 0.2 nm/s (DISCOVERY-635, DENTON), which was followed by a lift-off process. Finally, contact electrodes of the Hall-bar structures were fabricated using the method introduced above.

Using the fabricated Hall-bar structures, the electrical properties of 2DGFs and sputtered gold films were characterized by standard low-frequency measurements using a lock-in amplifier (SR 830, Stanford Research) and applying an alternating (*f* = 31 Hz) current with an amplitude of 100 μA from an AC Current Souce (6221, Keithley). For the measurement of the sheet resistance (as schematically shown in Supplementary Fig. 13), the voltage between electrodes 1 and 2 of the sample (*V*_12_) was first measured, the sheet resistance can then be calculated as ${R}_{S}=\frac{W}{L}\frac{{V}_{12}}{I}$, where *W* and *L* are the geometrical parameters defining the Hall-bar structure and *I* is the constant current through the Hall-bar.

### Measurement of nonlinear emission spectra

The setup for the measurement of the nonlinear emission spectrum of 2DGFs is schematically shown in Supplementary Fig. 15. For the SHG and THG measurements (Fig. 3b), laser pulses generated from a Ti:sapphire femtosecond laser (Mai Tai HP, Spectra-Physics) with a central wavelength of 1550 nm (~140-fs pulse width, 80-MHz repetition rate) was used for the excitation. A 20× objective (0.7 NA, Nikon) was used to focus the *p*-polarized incident pulses (800-mW average power) onto the samples (at a fixed incident angle of ~30° and a spot size of ~20 μm) and collect the reflective spectra of the nonlinear emission. After passing through a 950 nm short-pass filter blocking the reflected excitation laser pulses, the nonlinear emission was directed into a charge-coupled device (CCD) camera (DS-Fi3, Nikon) and a spectrometer (Shamrock SR-750, Andor) for imaging and spectral analysis, respectively. For the SHG and MPPL measurements (Fig. 3d), laser pulses generated from a Ti:sapphire femtosecond laser (Mai Tai HP, Spectra-Physics) with a central wavelength of 800 nm (~100-fs pulse width, 80-MHz repetition rate) were used for the excitation. A 50× objective (0.8 NA, Nikon) was used to focus the *p*-polarized incident pulses (5-mW average power) onto the samples (at a fixed incident angle of ~30° and a spot size of ~1 μm) and collect the reflective spectra of the nonlinear emission. After passing through a 700 nm short-pass filter blocking the reflected excitation laser pulses, the nonlinear emission was directed into a CCD camera and a spectrometer (QE Pro, Ocean Insight) for imaging and spectral analysis, respectively.

To avoid the impact of surface roughness on the nonlinear emission measurement, the 200-nm-thick sputtered gold film used for comparison was fabricated using a template stripping method to obtain excellent surface smoothness. Firstly, a gold film with a thickness of 200 nm was deposited onto a cleaned silicon substrate by magnetron sputtering at a base pressure of 5 × 10^−6^ Torr at a deposition rate of 0.2 nm/s. Secondly, a droplet (10 μL) of epoxy glue (EPO-TEK 301-2) was admitted onto the gold film, followed by the placement of a cleaned glass substrate on the top. Thirdly, the structure was transferred onto a hot plate to cure the epoxy under 80 °C for 3 h, and then was slowly cooled down to room temperature. Finally, the glass substrate was detached from the silicon substrate with a gold film having an atomically smooth surface attached to it.

### Simulation of nonlinear emission

To simulate thickness-dependent SHG and THG signals of 2DGFs, the eigen-state wave functions and eigen-energies were first calculated using the quantum electrostatic model^6^, which is based on the self-consistent solution of Schrödinger and Poisson equations. After that, a standard approach based on the perturbation theory for the intersubband transition (ISBT)-contributed nonlinearity is adopted to calculate the second- and third-harmonic susceptibilities (*χ*^*(*2)^ and *χ*^(3)^). Because the energy of the THG photons (2.4 eV) from 1550-nm pulse excitation is close to the interband transition (IBT) in gold, the contribution of IBT to *χ*^(3)^ was also calculated using the density matrix approach. To obtain the thickness-dependent SHG and THG intensities, experimental values of the incident pulse intensity as well as reflectivity at the fundamental, second- and third-harmonic wavelengths were used. Finally, all the intensities were normalized to the maxima (Supplementary Fig. 16). The simulated thickness-dependent SHG intensities with the contribution from ISBTs (Supplementary Fig. 16a) agree well with the experimental spectra (Fig. 3c, top panel). When contributions from both ISBTs (Supplementary Fig. 16b, sharp oscillations) and IBTs (Supplementary Fig. 16c, a broad peak) are considered to the simulated THG, the experimentally obtained THG thickness dependence (Fig. 3c, bottom panel) is well reproduced.

### Fabrication of nanoribbon arrays

Nanoribbon arrays based on 2DGFs were fabricated using the local etching approach introduced above. Nanoribbon arrays based on sputtered gold films were fabricated as follows. EBL was first used to define masks of the nanoribbon array structures on a mica substrate. Secondly, to avoid the use of metallic adhesion layers (such as Cr, Ti) that can introduce significant optical loss to plasmonic structures, the exposed region of the masks was functionalized with a monolayer of (3-aminopropyl)trimethoxysilane instead before the deposition of gold^43^. Then, gold films with various thicknesses were deposited on the substrates under a base pressure of ~5 × 10^−6^ Torr at a rate of 0.2 nm/s (DISCOVERY-635, DENTON), which was followed by a lift-off process to obtain the nanoribbon arrays.

### Numerical simulations for nanoribbon arrays

Transmission through gold nanoribbon arrays was numerically simulated using the finite element method (COMSOL Multiphysics software). The nanoribbon arrays were illuminated with a plane wave at normal incidence. Due to the invariance of the numerical problem in one of the directions, its dimensionality was reduced to 2D. Taking advantage of the symmetry of the structure, a unit cell of the array was modeled with periodic boundary conditions set on its sides. Perfectly matched layers were introduced at the top and bottom of the simulation domain to ensure the absence of back-reflection. The nanoribbon was set to have a rectangular cross-section with the width $w$ and thickness $t$, both of which were varied. In the case of 2DGFs, the single crystal permittivity from ref. ^57^ was taken, while in the polycrystalline case, data from ref. ^58^ was used. Additionally, to take into account additional losses related to the thickness-dependent electron scattering on the nanoribbon boundaries, the permittivities in both cases were corrected using the Fuchs theory^59^. The refractive index of a mica substrate was taken to be 1.53.

### Supplementary information

Supplementary Information

Peer Review File

## Data Availability

The data that support the findings of this study have been included in the main text and Supplementary Information. All other relevant data supporting the findings of this study are available from the corresponding authors upon request.
