# Supplementary material for: Large area single crystal gold of single nanometer thickness for nanophotonics
Source: Nat Commun. 2024 Apr 2;15:2840. doi: 10.1038/s41467-024-47133-7 (PMC10987654; doi:10.1038/s41467-024-47133-7)
Supplement: Supplementary file 1 — Supplementary Information [file 41467_2024_47133_MOESM1_ESM.pdf]

Supplementary Information: Large area single crystal gold of single nanometer thickness for nanophotonics

Chenxinyu Pan[1], Yuanbiao Tong[1], Haoliang Qian[2], Alexey V. Krasavin[3], Jialin Li[1], Jiajie Zhu[1], Yiyun Zhang[2], Bowen Cui[1], Zhiyong Li[1,4,5], Chenming Wu[1], Lufang Liu[1], Linjun Li[1,4,5], Xin Guo[1,4,5], Anatoly V. Zayats[3,], Limin Tong[1,6,*] and Pan Wang[1,4,5,*]*

[1]Interdisciplinary Center for Quantum Information, New Cornerstone Science Laboratory, State Key Laboratory of Extreme Photonics and Instrumentation, College of Optical Science and Engineering, Zhejiang University, Hangzhou 310027, China

[2]Interdisciplinary Center for Quantum Information, State Key Laboratory of Extreme Photonics and Instrumentation, ZJU-Hangzhou Global Scientific and Technological Innovation Center, Zhejiang University, Hangzhou 310027, China

[3]Department of Physics and London Centre for Nanotechnology, King's College London, Strand, London WC2R 2LS, UK

[4]Jiaxing Key Laboratory of Photonic Sensing & Intelligent Imaging, Jiaxing 314000, China

[5]Intelligent Optics & Photonics Research Center, Jiaxing Research Institute Zhejiang University, Jiaxing 314000, China

[6]Collaborative Innovation Center of Extreme Optics, Shanxi University, Taiyuan 030006, China

*Corresponding authors: a.zayats@kcl.ac.uk, phytong@zju.edu.cn, nanopan@zju.edu.cn

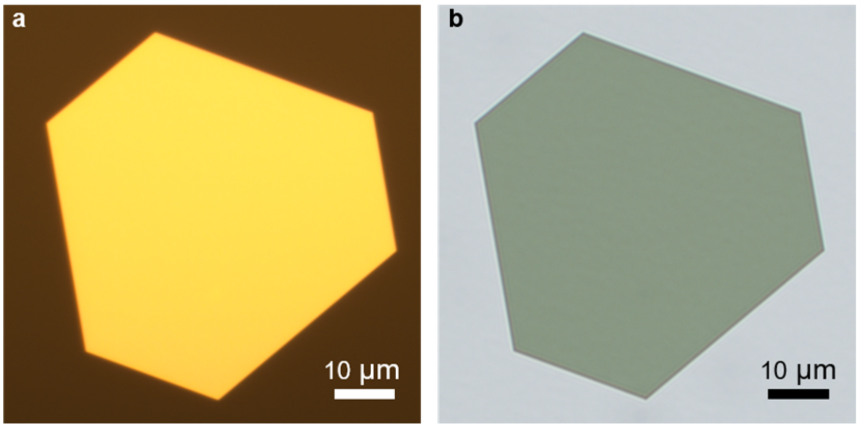

Supplementary Fig. 1 | Optical micrographs of a typical gold flake. a,**b**, Optical micrographs of a gold flake with a thickness of 30 nm taken in reflection (**a**) and transmission (**b**). Gold flakes were chemically grown on substrates using a modified wet-chemical method. By controlling the growth time, the thickness of the gold flakes can be tuned from ~10 to 100s of nanometers. Simutaneously, the lateral size of the gold flakes increses from ~10 to 100s of micrometers.

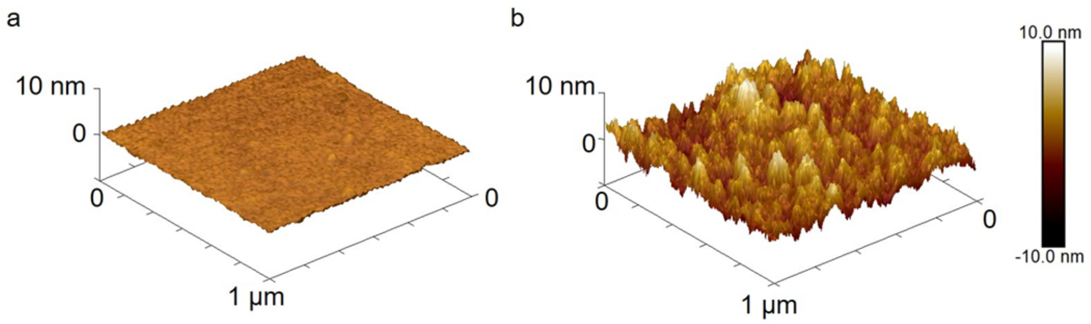

Supplementary Fig. 2 | Chemical etching a polycrystalline gold film. a,**b**, AFM images of the surface of a template-stripped polycrystalline gold film before (**a**) and after (**b**) the chemical etching. Due to the difference in the etching rate for gold with different crystalline facets, the surface of the template-stripped gold film became rough after the chemical etching.

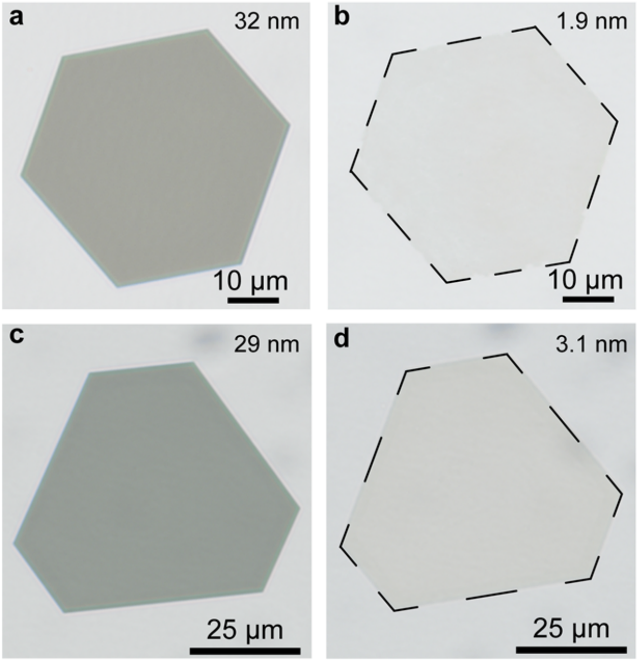

Supplementary Fig. 3 | Comparison of lateral sizes of gold flakes before and after chemical etching. a–d, Optical transmission micrographs of two different gold flakes taken before (**a**,**c**) and after (**b**,**d**) chemical etching. Micrographs shown in **a** and **b** are from the same as Fig. 1b. It is worth noting that the chemical etching process starts simultaneously from the top surface and edges of the gold flakes, but might proceed with a different etching rate due to the difference in the corresponding crystalline facets (as demonstrated in Supplementary Fig. 2). At the same time, it can be easily seen that the lateral size remaining almost unchanged after chemical etching, which is critical for the fabrication of 2D gold with a large area. The relatively thin thickness of the starting gold flakes is beneficial for obtaining 2DGFs with a minimal decrease in the lateral size.

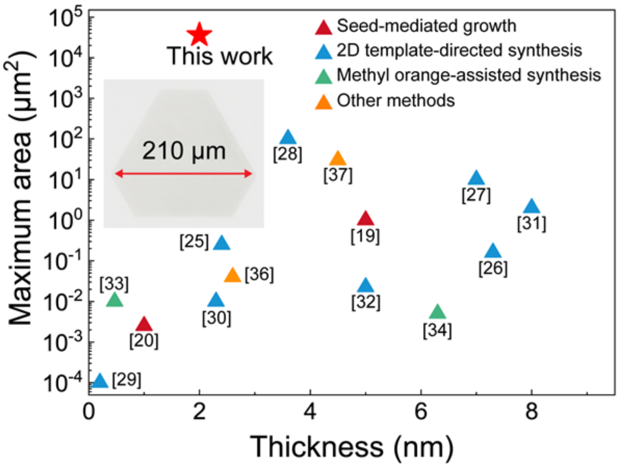

Supplementary Fig. 4 | Comparison of area and thickness of some representative 2D gold. Comparison of the area and thickness of a 2DGF (2 nm in thickness, 210 μm in lateral size, as shown in the inset) fabricated using the ALPE approach (red star) with counterparts obtained using other approaches (the corresponding references are given in the labels).

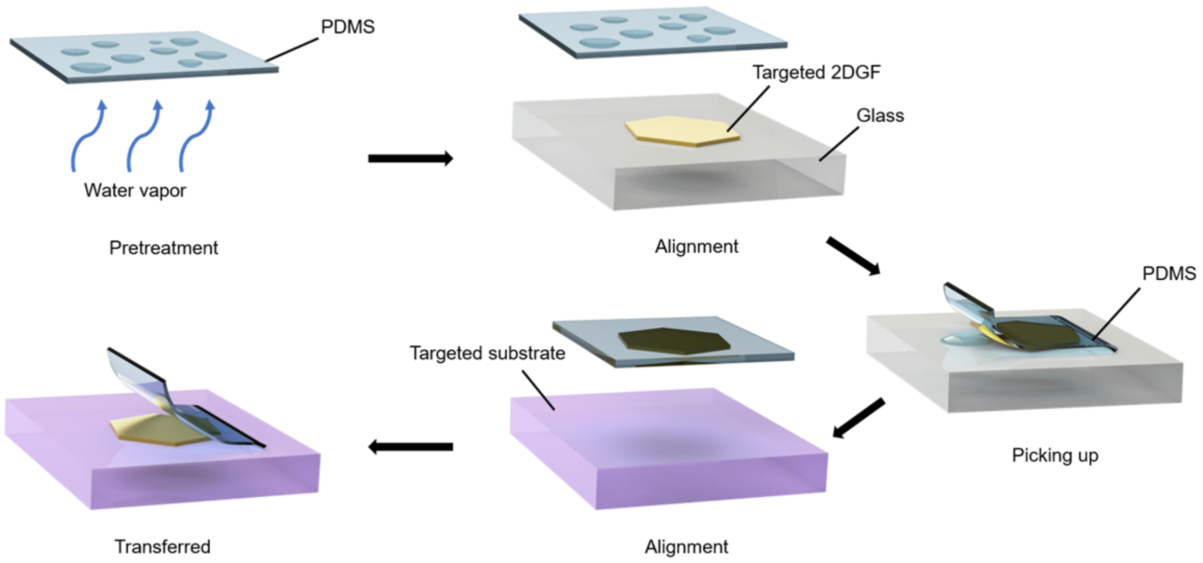

Supplementary Fig. 5 | Schematic diagram of PDMS-assisted transfer printing of a 2DGF onto a targeted substrate. A small patch of PDMS used for transfer printing of 2DGFs was first treated with water vapor to form numerous micrometer-scale water droplets on it. Then, under an optical microscope, the PDMS was quickly aligned (usually within a minute) and pressed onto the targeted 2DGF, then it was slowly withdrawn backward to pick the 2DGF up. Finally, the 2DGF attached on the PDMS film was aligned and made a full contact with the targeted substrate or structure. The 2DGF was left on the substrate or structure after a careful withdrawal of the PDMS film.

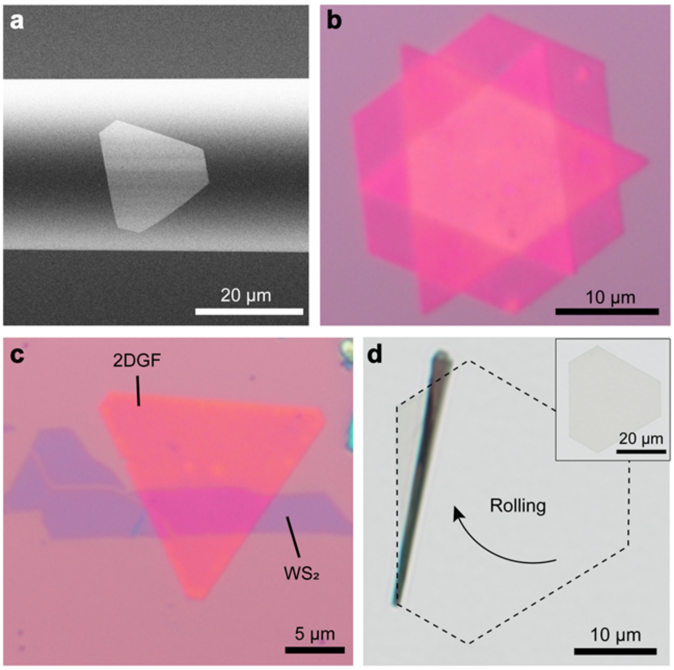

Supplementary Fig. 6 | Transfer printing of 2DGFs. a, Scanning electron microscopy image of a 2DGF (3.1 nm in thinckness) transfer-printed onto the sidewall of a 37-μm-diameter microfiber. **b**, Optical micrographs of a three-layer stack of 2DGFs (each ~3 nm in thickness) on a SiO_2/Si wafer. **c,** Optical micrograph of a 2DGF-WS_2 heterostructure. **d,** Optical micrograph of a rolled-up 3.6-nm-thick 2DGF. The dashed line shows the outline of the original 2DGF shown in the inset.

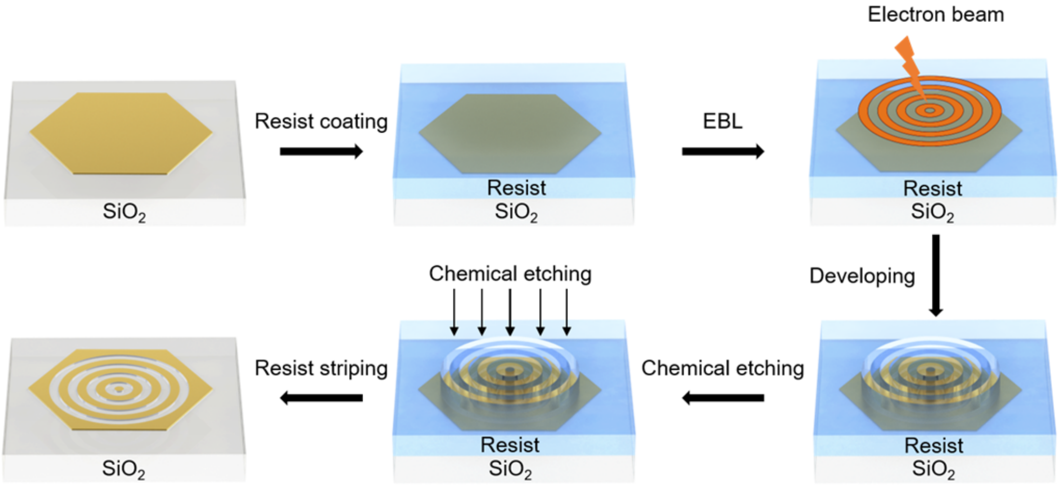

Supplementary Fig. 7 | Schematic illustration of processes for local etching of gold flakes. A gold flake on a substrate was first spin-coated with a 200-nm-thick resist and a 90-nm-thick conductive layer. Secondly, the resist on the gold flake was exposed with an electron beam to write the designed pattern, which was followed by a developing process to remove the exposed resist. Then, the sample was immersed into an etching solution to precisely etch the exposed gold. Finally, the resist was stripped off in acetone, and the sample was rinsed with ethanol and deionized water.

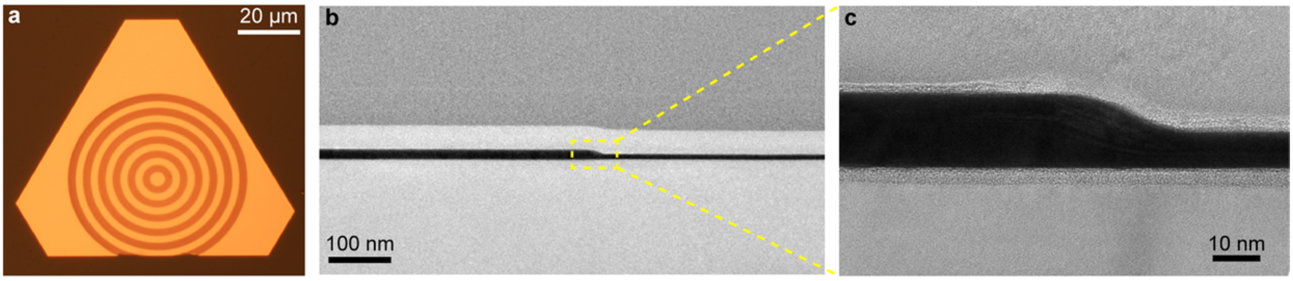

Supplementary Fig. 8 | Characterization of the etched edge of 2DGF. a, Optical reflection micrograph of a gold flake locally etched to implement a concentric ring pattern shown in Fig. 1f. **b**, Cross-sectional TEM image of an etched edge of the concentric ring pattern. **c**, Enlarged view of the marked region in **b**. It can be clearly seen that, at the etched edge, the thickness of the gold flake decreases gradually from 16 to 8 nm with a slope of ~30°. The gradual change in the thickness around the etched edge may be due to the non-wettability between the resist mask at the etched edge and the solvent, water. This can be improved by treating the resist mask with oxygen plasma to increase its wettability to water.

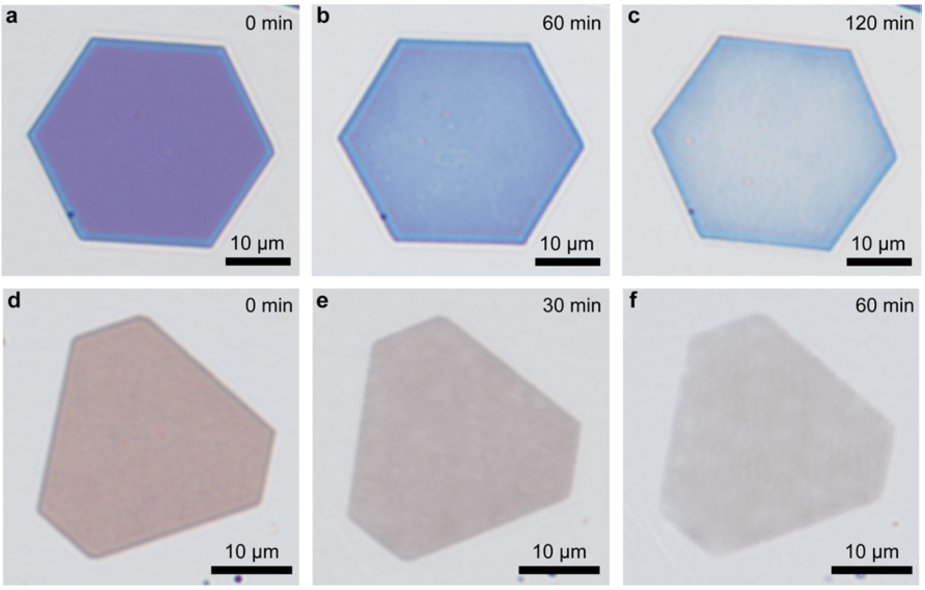

Supplementary Fig. 9 | Fabrication of 2D silver and copper flakes using ALPE approach. Optical transmission micrographs of silver (**a–c**) and copper (**d–f**) flakes taken at various etching times.

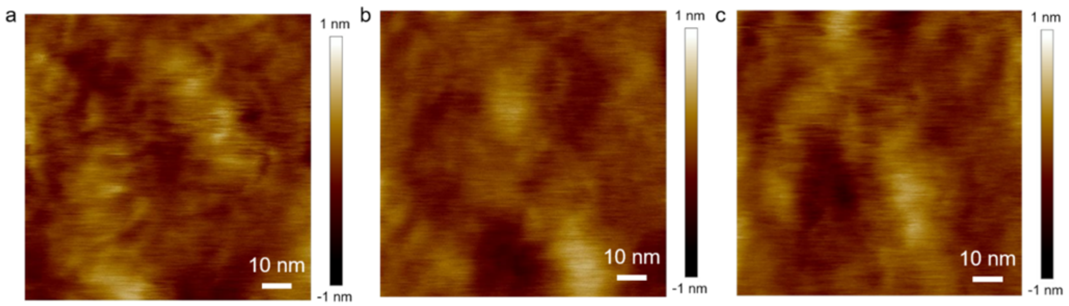

Supplementary Fig. 10 | Surface roughness of the 2DGF from Fig. 2a. a–c, Small-area (~0.01 μm^2) AFM images of three different locations on the 2DGF, which give RMS roughness of 0.154 nm (a), 0.152 nm (b) and 0.154 nm (c).

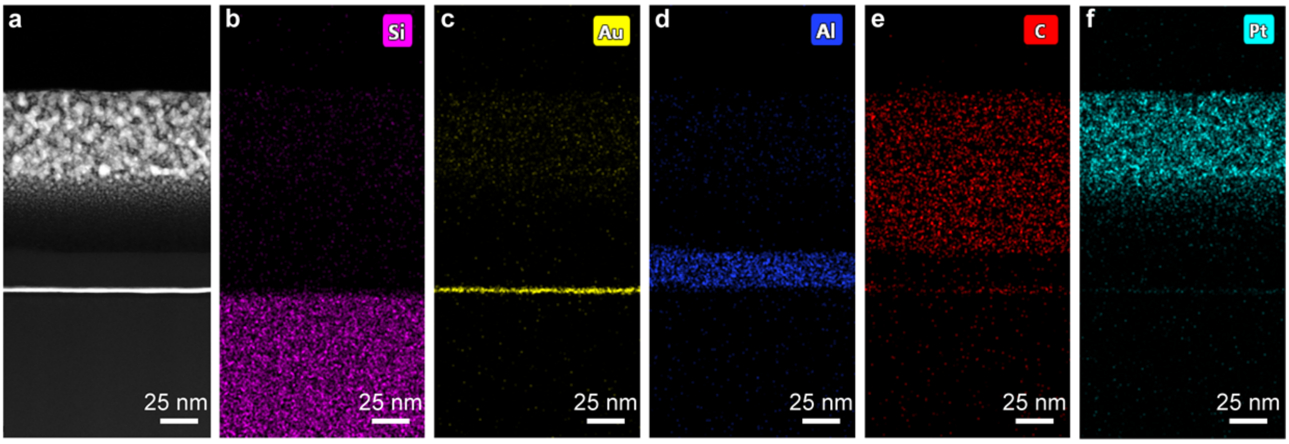

Supplementary Fig. 11 | Elemental mapping results obtained from the cross-section of the 2DGF shown in Fig. 2d. **a**, Cross-sectional TEM image of the 2DGF. **b**–**f**, Elemental maps of the sample presented in **a**, showing the distribution of silicon (**b**), gold (**c**), aluminum (**d**), carbon (**e**) and platinum (**f**).

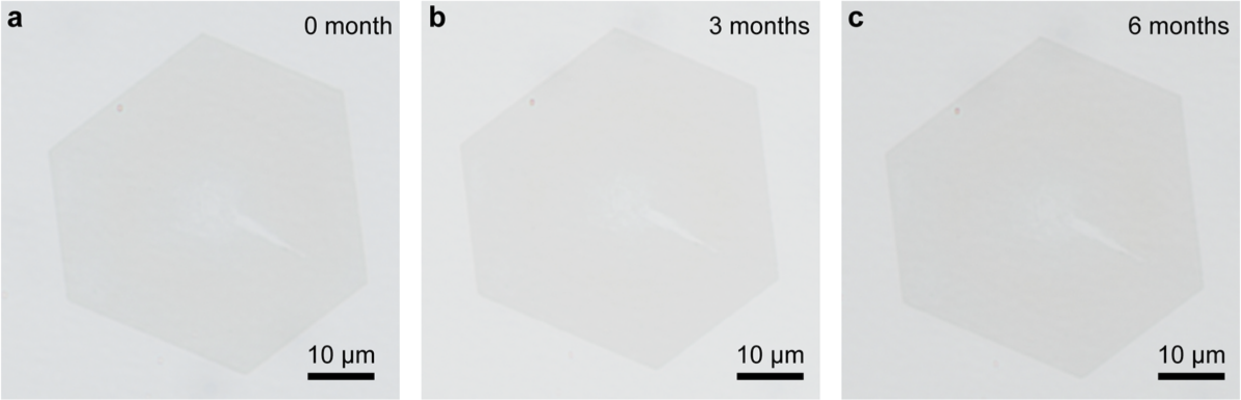

Supplementary Fig. 12 | Long-term stability test of 2DGFs. a, Optical micrograph of a freshly fabricated 2DGF with a thickness of 2.5 nm. **b**,**c**, Optical micrographs of the 2DGF taken after 3 months (**b**) and 6 months (**c**). The 2DGF was stored in ethanol to protect it from the contamination during the test. There is no observable change in the optical transmission micrographs after 6 months, indicating the excellent stability of the 2DGFs at room temperature.

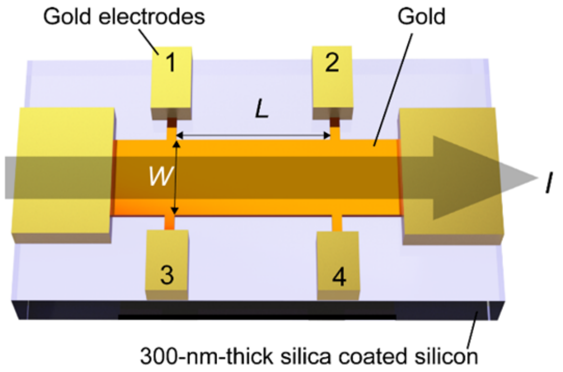

Supplementary Fig. 13 | Schematic illustration of the Hall-bar structure used for measurements of sheet resistance. For the measurement of sheet resistance, the voltage between electrodes 1 and 2 of the sample (V_{12}) was first measured, the sheet resistance can then be calculated as $R_S = \frac{W}{L}\frac{V_{12}}{I}$, where W and L are the geometrical parameters defining the Hall-bar structure and I is the constant current through the Hall bar.

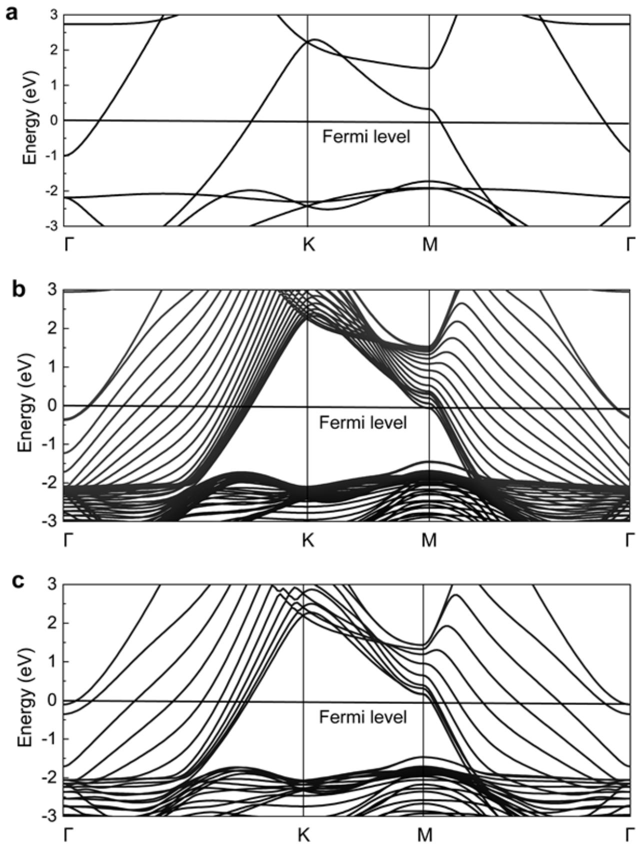

Supplementary Fig. 14 | Thickness dependence of electronic band structures of gold. a–c, Electron energy bands simulated for a bulk gold (**a**), a 20 atomic layer-thick gold slab (**b**) and a 10 atomic layer-thick gold slab (**c**) with a hexagonal close-packed structure. The calculations were performed using the all-electron full-potential APW + lo method as implemented in the WIEN2k code within the PBG-GGA functional. The experimental lattice constant of 4.08 Å was used for the calculations.

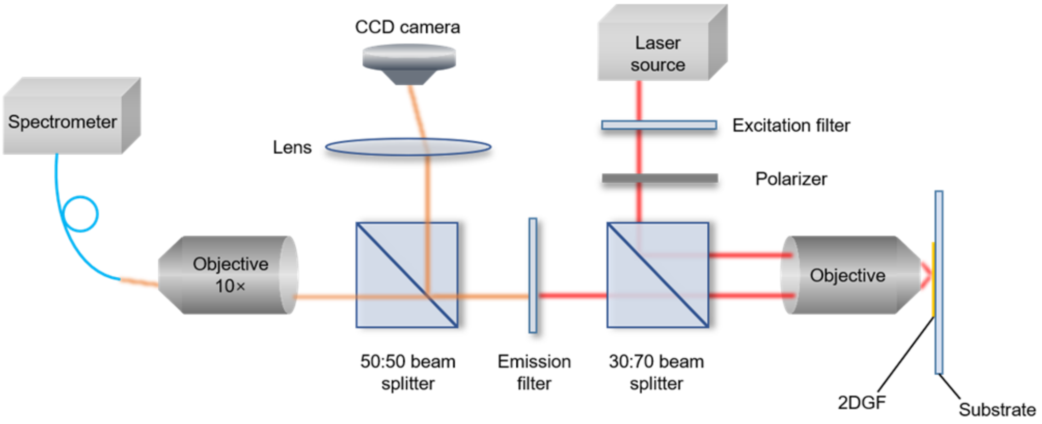

Supplementary Fig. 15 | Setup for measurements of nonlinear emission from 2DGFs. Collimated *p*-polarized femtosecond laser pulses used for the excitation after passing through an excitation filter were focused onto 2DGFs with an objective. The reflected nonlinear optical signal was collected by the same objective. After passing through an emission filter blocking the reflected excitation laser pulses, the nonlinaer emission was directed into a CCD camera and a spectrometer for imaging and spectral analysis, repectively.

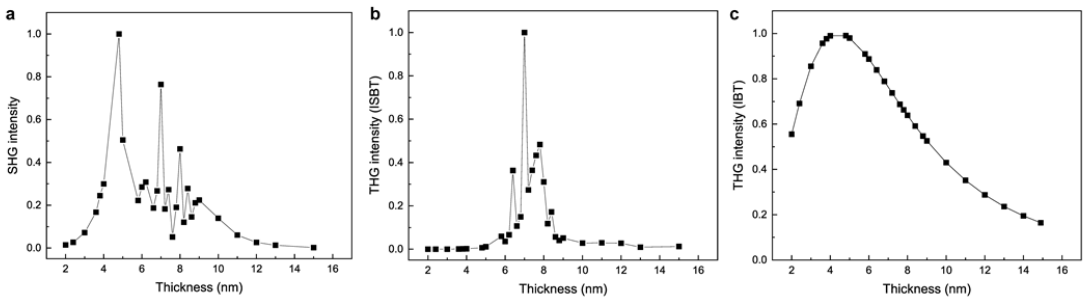

Supplementary Fig. 16 | Simulated SHG and THG intensities. a-c, Thickness-dependent SHG (**a**) and THG (**b**,**c**) intensities, simulated taking into account ISBTs (**a**,**b**) and IBTs (**c**). The simulated thickness-dependent SHG intensities with contribution from the ISBTs agree well with the experimental dependence (Fig. 3c, top panel). When both contributions for the THG from ISBTs (**b**) and IBTs (**c**) are considered, the experimentally obtained THG dependence is well reproduced.

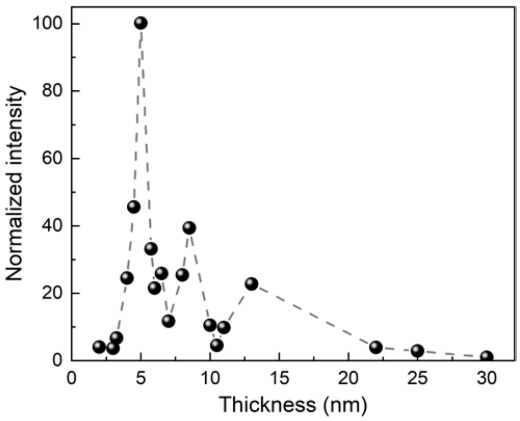

Supplementary Fig. 17 | Thickness-dependent SHG intensities from 2DGFs excited with 800-nm laser pulses. The dependence of SHG intensity on the thickness is extracted from the nonlinear emission spectra of 2DGFs with various thicknesses (e.g., partially shown in the inset of Fig. 3d) assuming incoherent addition of the SHG and MPPL signals, and normalized by the SHG intensity from a 30-nm-thick 2DGF.

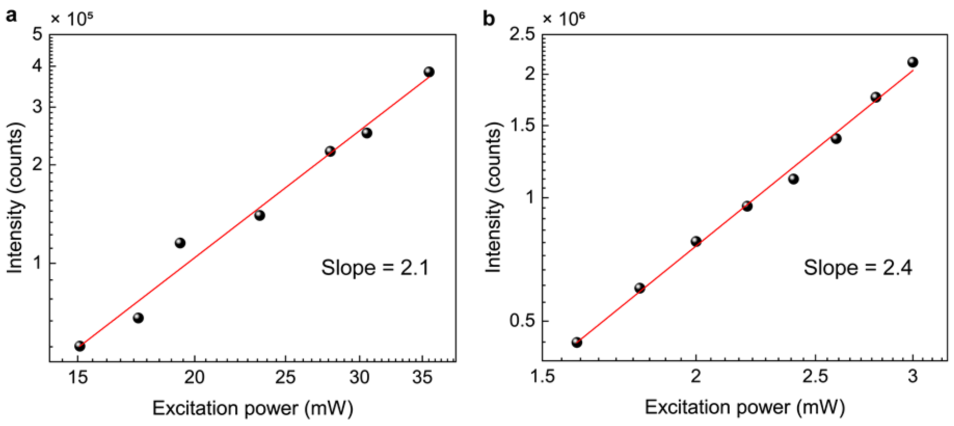

Supplementary Fig. 18 | Determination of nonlinear orders of MPPL from gold flakes. a,**b**, The dependence of spectrally-integrated intensities of MPPL from gold flakes with thicknesses of 19 (**a**) and 5 nm (**b**) on the excitation power. The spectrally-integrated MPPL intensities are extracted from the corresponding nonlinear emission spectra by assuming incoherent addition of SHG and MPPL signals. For the thickness of gold flake of 19 nm, a linear fit to the integrated MPPL intensities gives a slope of 2.1, indicating that the photoluminesce process is mainly due to two-photon photoluminesce. However, when the thickness of gold flake is decreased to, e.g., 5 nm, a slope of 2.4 is observed, showing the coexistence of two-photon and three-photon photoluminesce.

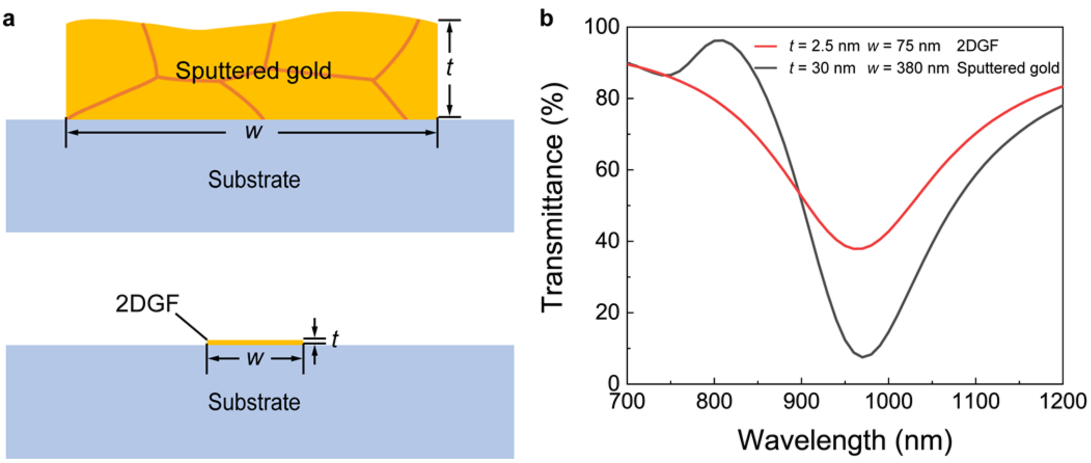

Supplementary Fig. 19 | Comparison of plasmonic properties of nanoribbons fabricated using a 2DGF and a sputtered gold film. a, Schematic illustrations of the nanoribbons based on a 30-nm-thick polycrystalline sputtered gold film and a 2.5-nm-thick single-crystal 2DGF. **b**, Simulated transmission spectra of nanoribbon arrays based on a 30-nm-thick sputtered gold film (black line) and a 2.5-nm-thick 2DGF (red line).

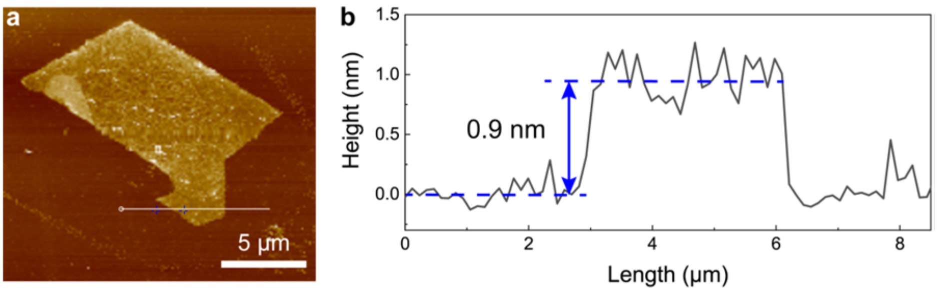

Supplementary Fig. 20 | Sub-nanometer 2DGF. **a**, AFM image of a sub-nanometer 2DGF. **b**, Line scan along the white line indicated in **a**, showing a gold thickness of ~0.9 nm, or ~5 atomic layers (cf. Fig. 2e). In principle, large area 2DGFs with thickness down to a single-atomic layer could be fabricated using the ALPE approach. However, due to the slight fluctuation in the thickness of the initial gold flake across its whole area and non-uniformity of the thickness of the native organic layer on the flake surface, it is challenging to obtain sub-nanometer 2DGFs with a large area. By further improvement of the thickness uniformity of the initial gold flake and removing the native organic layer, it is possible to push the thickness of 2DGF down to a single-atomic level.
